# Improving accuracy for stereotactic body radiotherapy treatments of spinal metastases

**DOI:** 10.1002/acm2.12395

**Published:** 2018-06-26

**Authors:** James Rijken, Barry Jordan, Scott Crowe, Tanya Kairn, Jamie Trapp

**Affiliations:** ^1^ Genesis Care Flinders Private Hospital Bedford Park SA Australia; ^2^ Queensland University of Technology Brisbane QLD Australia; ^3^ Royal Brisbane and Women's Hospital Brisbane QLD Australia

**Keywords:** accuracy, control point, SABR, spine, VMAT

## Abstract

**Purpose:**

Use of SBRT techniques is now a relatively common recourse for spinal metastases due to good local control rates and durable pain control. However, the technique has not yet reached maturity for gantry‐based systems, so work is still required in finding planning approaches that produce optimum conformity as well as delivery for the slew of treatment planning systems and treatment machines.

**Methods:**

A set of 32 SBRT spine treatment plans based on four vertebral sites, varying in modality and number of control points, were created in Pinnacle. These plans were assessed according to complexity metrics and planning objectives as well as undergoing treatment delivery QA on an Elekta VersaHD through ion chamber measurement, ArcCheck, film‐dose map comparison and MLC log‐file reconstruction via PerFraction.

**Results:**

All methods of QA demonstrated statistically significant agreement with each other (*r* = 0.63, *P* < 0.001). Plan complexity and delivery accuracy were found to be independent of MUs (*r* = 0.22, *P* > 0.05) but improved with the number of control points (*r* = 0.46, *P* < 0.03); with use of 90 control points producing the most complex and least accurate plans. The fraction of small apertures used in treatment had no impact on plan quality or accuracy (*r* = 0.29, *P* > 0.05) but rather more complexly modulated plans showed poorer results due to MLC leaf position inaccuracies. Plans utilizing 180 and 240 control points produced optimal plan coverage with similar complexity metrics to each other. However, plans with 240 control points demonstrated slightly better delivery accuracy, with fewer MLC leaf position discrepancies.

**Conclusion:**

In contrast to other studies, MU had no effect on delivery accuracy, with the most impactful parameter at the disposal of the planner being the number of control points utilized.

## 1 INTRODUCTION

Stereotactic body radiotherapy (SBRT) describes extracranial treatment techniques which utilize a larger delivery of radiation dose than conventional radiotherapy and in fewer fractions, resulting in a higher biological effective dose for the treatment site.^1^ As with stereotactic radiosurgery for brain metastases,^2^ the SBRT technique is able to provide extra dose to the target volume without exceeding recommended normal tissue tolerances. In the case of spinal metastases, the spinal cord may have already been irradiated through conventional radiotherapy and SBRT provides a noninvasive treatment option.^3^

In order to limit dose to the spinal cord, modulated treatment techniques are used to produce complex dose distributions that spare organs at risk.^4^, ^5^, ^6^ SBRT spine treatment can be delivered using a number of treatment machines like helical tomotherapy units, robotic radiosurgery systems or on a linac utilizing intensity‐modulated radiotherapy (IMRT) or volumetric‐modulated arc therapy (VMAT). In an Eclipse treatment planning system (TPS) & Varian linac environment, single arc VMAT has been found to produce inferior target coverage and normal tissue sparing compared to IMRT, but two arc VMAT is able to produce comparable results.^7^ Comparisons between flattening filter free (FFF) and conventional modalities have demonstrated significant improvements in normal tissue/spinal cord sparing for FFF plans with a greater number of control points, while achieving the same level of target coverage.^8^, ^9^ While these and other planning studies have been conducted for SBRT spine, there are still gaps in the literature regarding the overall optimization of the SBRT spine technique, which requires further investigation.

A couple of planning studies have also included quality assurance (QA). A recent study found accuracy improvements for an individual SBRT spine case when the gantry spacing between control points was decreased from 4° to 3°.^10^ The delivery accuracy of different IMRT and VMAT techniques has also been investigated for spinal treatment.^11^ IMRT was measured to deliver more accurately than VMAT due to its reduced complexity. VMAT beams can be delivered more accurately if optimized with a monitor unit (MU) limit, which can produce a less complex plan. However, these findings may be unique to the dose calculation and optimization algorithms of the Eclipse TPS and the accuracy of the Varian linac setup. The study also allowed a slightly higher cord dose which could make large impacts in plan conformity. Another recent study investigated the effect of high definition MLCs on plan quality, complexity and deliverability^12^ and found that while slight improvements to quality could be made for VMAT SBRT spine treatments, plan complexity increased and thus delivery accuracy suffered.

There is an overall lack of studies which combine both planning optimizations and QA results for SBRT spine treatment. This study aims to address this and improve upon previous studies through utilization of four different methods of QA. It is important to fully assess inaccuracies/accuracies in the TPS, so that it can be used confidently in the correct range of parameters.

## 2 MATERIALS AND METHODS

Four previously treated and anonymized SBRT spine CT and contour datasets were selected with differing vertebra and target volume shapes (targeting had been previously performed by radiation oncologists according to international guidelines,^3^, ^13^ CT was performed with 2 mm slices). Eight different SBRT spine plans were created on each CT image dataset, utilizing both the 6 MV and 6 MV FFF beam. Control points were set as planning constraints before inverse optimization and plans of 50, 90, 180, and 240 control points were created (Table 1). The 50 control point plans consisted of nine beams delivering IMRT, while the 90, 180, and 240 control point plans were 1, 2, and 2 arc VMAT plans, respectively. Plan complexity was measured through different metrics: (a) modulation index (MI) which considers the fluence map (higher MI implies higher complexity),^14^ (b) modulation complexity score (MCS) which considers aperture area variability and leaf sequence variability (higher MCS implies lower complexity),^15^ and (c) the small aperture score (SAS) which considers the fraction of fields used <10 mm (higher SAS corresponds to more small fields).^16^

**Table 1:** List of SABR spine treatment plans

Vertebral region	Planning volume	Energy	Control points
T12	Vertebral body	6 MV	50, 90, 180, 240
		6 MV FFF	50, 90, 180, 240
L2	Whole vertebra	6 MV	50, 90, 180, 240
		6 MV FFF	50, 90, 180, 240
L1	Vertebral body	6 MV	50, 90, 180, 240
		6 MV FFF	50, 90, 180, 240
T11	Whole vertebra	6 MV	50, 90, 180, 240
		6 MV FFF	50, 90, 180, 240

The TPS utilized was Pinnacle3^®^ 9.10 (Koninklijke Philips N.V., Amsterdam, The Netherlands). Plans were created within the TPS each with the same fractionation and dose constraints currently used clinically: 30 Gy in 3 fractions, with 100% of planned target volume (PTV) to be covered by 80% of prescribed dose,^17^ but ideally covered by 90% as per RTOG 0631.^13^ The other main metric concerned was assessment of the Paddick conformity index (CI) for target coverage (CI <1.0 implies poorer conformity)^18^ as per ICRU 91.^19^ Other departmental metrics concerned were PTV mean dose below 110% of prescription and 95% of the clinical target volume (CTV) to receive 98% of the prescription^1^ (CTV‐PTV margin of 2 mm). However, critical nervous structure (CNS) constraints always took priority. The main CNS structure concerned was the thecal sac with a maximum dose of 20 Gy.^20^

Each of the 32 clinical treatment plans created was given the same iteration schedule for inverse planning. The dose calculations were made using the collapsed cone convolution (CCC) algorithm on a 2 mm dose grid^21^ with a minimum leaf separation of 0.5 cm. The optimization schedule began with an initial 80 iterations after which the plan was assessed and the optimization objectives adjusted in order to achieve the desired conformity. After these adjustments, the plan was then optimized for a further 30 iterations. The plan was adjusted and optimized another two times, bringing the total number of iterations to 170 with three adjustments.

The treatment plans were delivered on an Elekta VersaHD^®^ linac (Elekta, Stockholm, Sweden) commissioned down to field sizes of 1 × 1 cm^2^ with a RAZOR™ Chamber (IBA Dosimetry GmbH, Schwarzenbruck, Germany) and an EDGE Detector™ (Sun Nuclear Corp., Melbourne, FL, USA). The first mode of QA was through isocenter ion chamber (IC) measurement in a solid water slab using the calibrated RAZOR™ Chamber (calibration traceable to primary standards laboratory). The dose measured by the small volume IC was compared to the TPS calculation. Second, the plans were delivered to an ArcCheck^®^ device (Sun Nuclear Corp., Melbourne, FL, USA). Third, film QA was performed in the sagittal plane, utilizing the ArcCheck as a phantom. The film employed was Gafchromic™ EBT3 (Ashland Specialty Ingredients, Bridgewater, NJ, USA) and was scanned on an Expression™ 10000XL scanner (Epson^®^, North Ryde, NSW, Australia) and processed through SNC Patient Software 6.1 (Sun Nuclear Corp., Melbourne, FL, USA). A film‐dose calibration was also performed at the time of delivery such that SNC Patient was able to handle exposures between 1 and 20 Gy. The dose maps derived from the film (red channel, based on the work of Micke et al.^22^) and the ArcCheck were then compared to respective dose maps from the TPS through gamma analysis in SNC Patient with respective gamma criteria of 3%/1.5 mm and 2%/2 mm. All gamma analyses in this study were performed through absolute dose comparison with a local calculation and a 10% low dose cut‐off threshold.^23^

Finally, QA on the treatment plan delivery was also performed through the PerFraction™ software package (Sun Nuclear Corp., Melbourne, FL, USA). PerFraction takes the MLC log‐files from the linac and reconstructs the dose delivered on the CT image dataset using its own CCC style algorithm. A gamma analysis of the two calculated dose distributions was performed (1%/1 mm) for each plan. Correlation of data was assessed using the Pearson correlation coefficient, *r*,^24^ given by

$$r=\frac{\sum_{i=1}^{n}\left(x_{i}-\overset{¯}{x}\right)\left(y_{i}-\overset{¯}{y}\right)}{\sqrt{\sum_{i=1}^{n}{\left(x_{i}-\overset{¯}{x}\right)}^{2}}\sqrt{\sum_{i=1}^{n}{\left(y_{i}-\overset{¯}{y}\right)}^{2}}}$$

where *n* is the number of samples and *x*_*i*_ and *y*_*i*_ are the samples. The statistical significance of any correlation was assessed through *P* values calculated using a Student's *t* test (*α *= 0.05).

## 3 RESULTS

**Figure 1:**
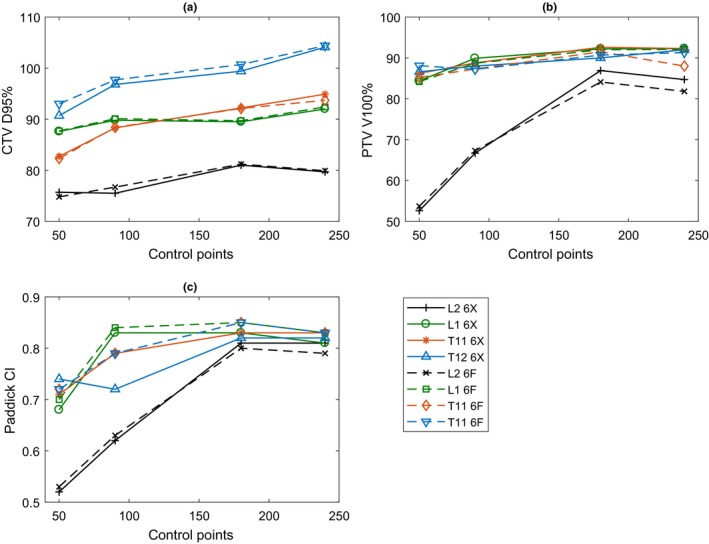
Plan quality metrics plotted against number of control points: (a) CTV D95%, (b) PTV V100%, and (c) Paddick CI.

Plans are labeled in the results according to their site (L1, L2, T11, or T12) and/or control points (50, 90, 180, or 240) and/or modality (“X” for 6 MV or “F” for 6 MV FFF). Plan quality results of the SBRT spine plans can be seen in Fig. 1. The most important planning criterion was the CNS dose constraint with the resultant median thecal sac dose being 18.7 Gy. The mean Paddick CI for 6 MV plans was 0.76 ± 0.09 and for 6 MV FFF was 0.77 ± 0.09. The impact of prioritizing CNS sparing can be seen in the difficulty to achieve good conformity and coverage (median CTV D95% was 89.9%, median PTV V100% was 88.0%, median PTV mean dose was 110.8%) in most cases. However, as the number of control points was increased, the coverage and conformity consistently improved (*r *=* *0.70, *P *<* *0.001). There was no significant correlation between plan quality and the number of MUs (*r *=* *0.16, *P *>* *0.05).

**Figure 2:**
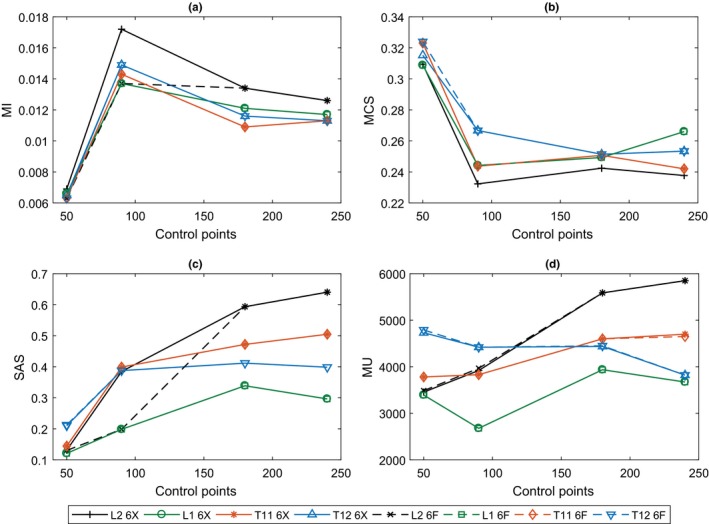
Plan metrics for each plan plotted against the number of control points: (a) MI, (b) MCS, (c) SAS, and (d) MU.

MCS and MI had no significant trend with MU (*r *=* *0.22, *P *>* *0.05). However, Fig. 2 shows both varied with the number of control points, demonstrating the same changes in plan complexity. The 50 control point IMRT plans were the least complex and the 90 control point VMAT were the most, with 180 and 240 being of comparable complexity. SAS increased with the number of control points used, with 180 and 240 control point plans having comparable SAS in most plans. No consistent trend was found between MU and the number of control points.

**Figure 3:**
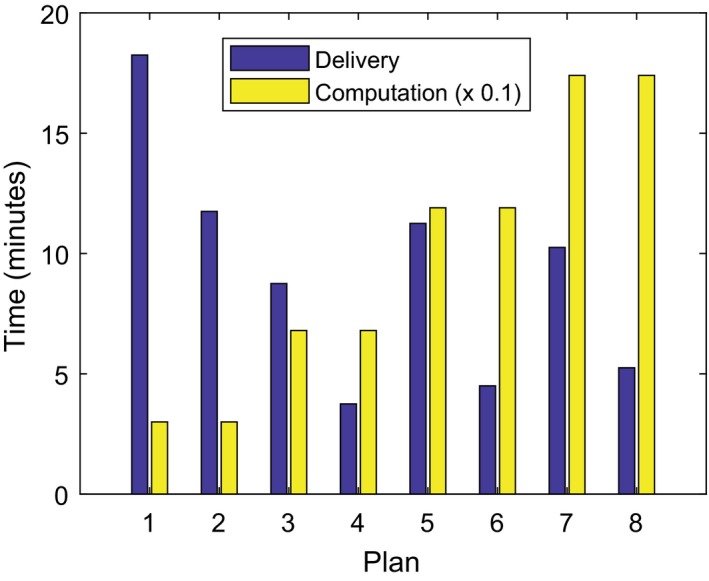
Average times taken for treatment planning and delivery for each plan type: (a) 50X, (b) 50F, (c) 90X, (d) 90F, (e) 180X, (f) 180F, (g) 240X, (h) 240F.

Average planning computation times and treatment delivery times are shown in Fig. 3. Computation times scale according to the number of control points utilized. Plan delivery times were quicker for FFF due to the higher dose rate and increased with the number of arcs or beams used.

**Figure 4:**
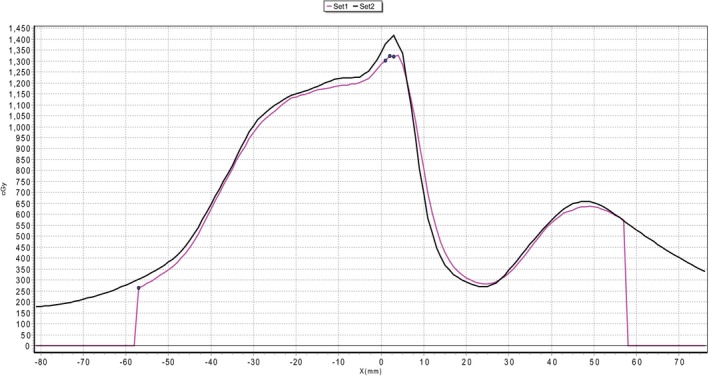
Film QA profile from the T11 240X plan with delivered dose (set 1) and planned dose (set 2). Gamma pass rate of 98.2% (3%/1.5 mm).

**Figure 5:**
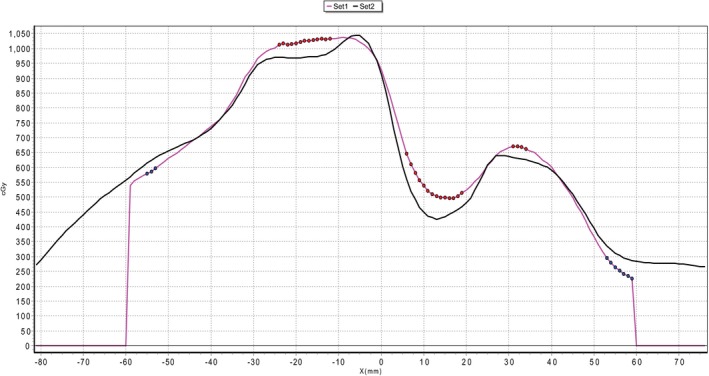
Film QA profile from the L2 90X plan with delivered dose (set 1) and planned dose (set 2). Gamma pass rate of 80.3% (3%/1.5 mm).

As an example, a plan that was delivered with a high level of accuracy was T11 240X with its film QA shown in Fig. 4, while a particularly poorly delivered plan, L2 90X, is shown in Fig. 5.

**Figure 6:**
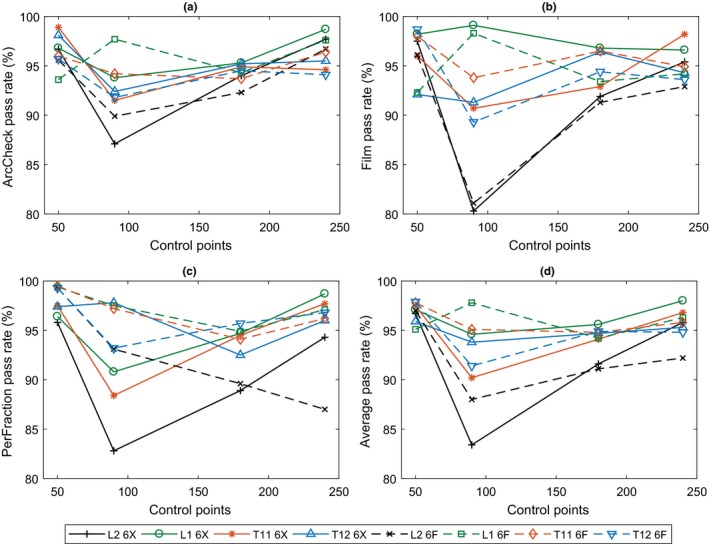
Gamma pass rates from QA for each plan through (a) ArcCheck (2%/2 mm), (b) film (3%/1.5 mm), (c) PerFraction 3D analysis (1%/1 mm), and (d) mean gamma pass rates plotted against the number of planned control points.

ArcCheck, Film, PerFraction, and mean pass rates plotted against control points are shown in Fig. 6. Film results would indicate that there is no consistent difference between 240 and 180 control point plans but combined with ArcCheck and PerFraction results, a statistically significant improvement in gamma pass rates was observed from 90 to 240 control points (*r *=* *0.46; *P *<* *0.03). The median respective pass rates for ArcCheck, Film, and PerFraction were 95.1%, 94.4%, and 95.9% at their respective gamma criteria. All methods of gamma analysis demonstrated statistically significant agreement with each other (*r *=* *0.63, *P *<* *0.001) with no such trend observed between pass rate and MUs (*r *=* *0.18, *P *>* *0.05). 50 and 240 control point plans were found to be the most accurate, with plans of 90 control points performing the most poorly and with the largest variation in results.

**Figure 7:**
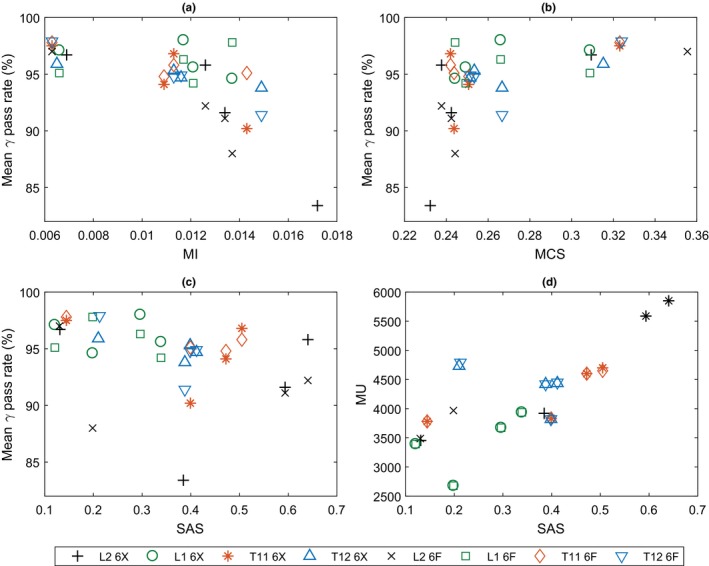
Plan delivery accuracy as a measure of mean gamma pass rate plotted against (a) MI, (b) MCS, and (c) SAS. (d) shows the relationship between MU and SAS.

Figure 7 shows the relationship between plan accuracy and plan complexity. (a) and (b) demonstrate that as plan complexity decreases, gamma rates become consistently better (*r *=* *0.51, *P *<* *0.02). (c) shows the lack of correlation between gamma pass rates and SAS for each plan (*r *=* *0.29, *P *>* *0.05) and (d) shows the relationship between SAS and MUs, demonstrating a statistically significant trend (*r *=* *0.79, *P *<* *0.001).

MCS and MI had no significant trend with MU (*r *=* *0.22, *P *>* *0.05) and while the median IC result was a −0.05% difference to TPS, isocenter dose differences became more negative as MU increased (*r *=* *0.46, *P *<* *0.01). Correlation data have been collated in Table 2.

**Table 2:** Pearson correlation coefficients and statistical significance between experiment data

Variable 1	Variable 2	*r*	*P*	Comment
Plan quality	MU	0.16	>0.05	No significant correlation
Plan quality	Control points	0.70	<0.001	Significant correlation
Plan complexity	MU	0.22	>0.05	No significant correlation
*γ* pass rate	MU	0.18	>0.05	No significant correlation
*γ* pass rate	SAS	0.15	>0.05	No significant correlation
*γ* pass rate	Control points (90–240)	0.46	<0.03	Significant correlation
*γ* pass rate	Plan complexity	0.51	<0.002	Significant negative correlation
MU	Plan complexity	0.51	<0.002	Significant negative correlation
*γ* pass rate	SAS	0.29	>0.05	No significant correlation
MU	SAS	0.79	<0.001	Significant correlation
Isocenter dose	MU	0.46	<0.01	Significant negative correlation
ArcCheck pass rates	Film pass rates	0.75	<0.001	Significant correlation
ArcCheck pass rates	PerFraction pass rates	0.58	<0.001	Significant correlation
Film pass rates	PerFraction pass rates	0.56	<0.001	Significant correlation

## 4 DISCUSSION

Given the CNS prioritization, it is not surprising that very few plans achieve the target goals (Fig. 1). However, such plans are still clinically deliverable as 27 Gy in 3 fractions. Contrary to other studies, no difference was found in conformity between 6 MV and 6 MV FFF plans,^9^ with IMRT plans consistently less conformal compared to 90 control point VMATs and poorer still compared to 180 and 240.^7^ No correlation could be found between the metrics of plan complexity and quality. These metrics are not under direct control of the user anyway, apart from SAS which can be influenced in the model. The most impactful parameter at the disposal of the planner is the number of control points utilized, with major improvements made to plan quality and consistency of quality. Plans with 50 control points composed the worst plans with their poor conformity attributed to the use of IMRT and its limitations.^25^, ^26^ No statistically significant trend could be observed between conformity and MUs, which is in disagreement with results shown by Kairn et al. for the Varian/Eclipse setup, where VMAT plans optimized with MU limitations resulted in poorer conformity.^11^

It can be seen that better quality plans only consistently resulted from increases to the number of control points (90 to 240), so it is of interest then that as control points increased, the likelihood of producing a plan with a greater SAS also increased [Fig. 2(c)]. Without fully analyzing the impact of SAS on plan quality, one might be tempted to deduce that small fields produce better quality plans — this was found not to be the case (*r *=* *0.15, *P *>* *0.05). The most complex plans resulted from setting control points to 90, causing the VMAT optimization algorithm to produce more complicated MLC shapes in order to meet planning objectives. IMRT plans were not as complex, even though there were fewer control points, due to optimization algorithm differences and as such, the delivery accuracy was high.

The relative improvements in treatment times are similar to those seen in other studies,^8^, ^27^, ^28^, ^29^ but not so severe as those shown by Kairn et al. where IMRT plans took around three times as long to deliver.^11^ This is the first report of average TPS computation times for a SBRT spine study.

The significant agreement between gamma pass rates from three methods of QA (Fig. 6) may be intuitive for comparisons between film and ArcCheck results (if one has refined their QA procedure), but PerFraction considers the MLC log‐files when assessing plan delivery, so the agreement is a little more subtle. The correlation, then, between ArcCheck/film and PerFraction is of interest and has not yet been reported for any MLC log‐file‐based system for SBRT spine. Past investigations of PerFraction have been limited to its EPID dosimetry capabilities^30^, ^31^, ^32^ and some work has been performed on other MLC log‐file‐based systems, demonstrating good agreement between conventional QA and MLC log‐file‐based patient QA.^33^, ^34^, ^35^, ^36^ Discrepancies have been demonstrated between actual and intended MLC leaf delivery for low MU control points, with good agreement shown between MLC log‐file and diode array assessment methods.^37^ A similar effect, in this study, may be attributed to the correlation between PerFraction and ArcCheck/film, thus inferring that losses in plan accuracy are due to MLC movement discrepancies. While most of the PerFraction gamma fail points were in peripheral tissue and neither consistently higher nor lower in dose, target and CNS doses were generally greater than the TPS. This may just indicate differences between the two dose computation algorithms’ treatment of bone — differences which have been demonstrated previously for other TPSs.^38^, ^39^ PerFraction's log‐file analysis thus shows promise as a QA tool to detect plans that cannot be delivered accurately, even for complicated treatments such as SBRT spine.

No statistically significant trends could be found between treatment delivery accuracy and MUs, which is in contrast to findings by Kairn et. al. where limiting MU substantially increased VMAT plan accuracy and deliverability.^11^ This may be due to differences in the optimization algorithms between Eclipse and Pinnacle. In any case, MU cannot be directly controlled in the TPS during inverse planning, so cannot be used as a tool to produce an optimum plan. It was found that plans with greater MU had treatment deliveries with a more negative dose difference at the isocenter. While MU had no impact on plan complexity, there was definite trend with SAS [Fig. 7(d)]. The relationship between MU and SAS makes rational sense: if the prescription is the same and the target volumes are comparable, plans with greater MUs must employ smaller fields. The lack of correlation between MUs and control points means MUs cannot be indirectly influenced by planners in the TPS.

Increasing from 180 to 240 control points (from 4° to 3° between control points), produced consistently better results [Fig. 6(d)] in agreement with the literature.^10^ It was shown previously that 90 control point plans were the most complex, and while this had no impact on the plan coverage/conformity, it did have an impact on accuracy/deliverability. Figures 7(a) and 7(b) show that as plan complexity increases, gamma pass rates become consistently worse. This trend has been shown in other studies also^11^, ^12^, ^16^ and is most likely due to MLC leaf position discrepancies becoming more frequent^37^ rather than model deficiencies.

As expected, plan complexity had no statistically significant impact on isocenter point dose measurements, as dose point placement followed the principles of ICRU 50 to ensure accuracy.^40^ SAS had no effect on the gamma pass rates in contrast to the previous studies concerning IMRT plans in Eclipse.^16^, ^41^ This may be due to optimization differences between TPSs or due to the fact that, while a greater SAS corresponded with plans delivered with less dose compared to the TPS (according to the isocenter measurement), the effect was not great enough to be noticed by ArcCheck or film gamma criteria. Small fields may not be modeled perfectly, as is often the case when using a single TPS model for all field sizes,^42^ but since the effect of SAS is so small it is not recommended for the physicist to spend too much time tweaking this in the model as an avenue of improving their department's SBRT spine accuracy. This study has shown that if the MLC model parameters have been sufficiently validated, accuracy improvements come from producing less complex plans, which is influenced by control points, a parameter that can be easily changed. The planner should feel confident that a less complex plan does not necessarily mean a plan with poorer coverage or conformity, as demonstrated by the lower complexity and higher conformity of plans with 240 control points.

Bringing all these concepts together, only one plan failed ArcCheck and film QA: L2 90X (Fig. 5). This plan had the lowest MCS (0.232) and the highest MI (0.0172), indicating that it was by far the most complex plan produced by the TPS in this study. It was then the most likely to have MLC leaf discrepancies, which is further corroborated by PerFraction showing the lowest pass rate of all 32 plans (82.8%). Through accuracy optimizations of the SBRT spine treatment technique, one could detect such a plan even before treatment QA or, better yet, avoid producing such a plan altogether.

## 5 CONCLUSION

A planning and QA study of 32 SBRT spine plans, optimized on four vertebral sites, was conducted in order to find optimum conditions for plan conformity and accuracy of delivery. Plan complexity metrics were found to be independent of plan quality, while QA through four independent methods demonstrated that plan conformity was influenced by the number of control points. In contrast to other studies, it was not MU that improved delivery accuracy but, rather, control points. Like studies on Varian/Eclipse systems, more complex plans were less accurate to deliver, which is attributed to MLC leaf position discrepancies, not solely small fields. The optimum SBRT spine plans in terms of both plan conformity and coverage as well as plan delivery accuracy came from increasing control points to 240.

## CONFLICTS OF INTEREST

The authors have no other relevant conflicts of interest to disclose.
